# Importance of Cough and *M. tuberculosis* Strain Type as Risks for Increased Transmission within Households

**DOI:** 10.1371/journal.pone.0100984

**Published:** 2014-07-02

**Authors:** Edward C. Jones-López, Soyeon Kim, Geisa Fregona, Patricia Marques-Rodrigues, David Jamil Hadad, Lucilia Pereira Dutra Molina, Solange Vinhas, Nancy Reilly, Stephanie Moine, Soumitesh Chakravorty, Mary Gaeddert, Rodrigo Ribeiro-Rodrigues, Padmini Salgame, Moises Palaci, David Alland, Jerrold J. Ellner, Reynaldo Dietze

**Affiliations:** 1 Section of Infectious Diseases, Department of Medicine, Boston Medical Center and Boston University School of Medicine, Boston, Massachusetts, United States of America; 2 Department of Preventive Medicine and Community Health, New Jersey Medical School, Rutgers, The State University of New Jersey, Newark, New Jersey, United States of America; 3 Núcleo de Doenças Infecciosas (NDI), Universidade Federal do Espírito Santo (UFES), Vitória, Brazil; 4 Mycobacteriology Laboratory, Núcleo de Doenças Infecciosas, UFES, Vitória, Brazil; 5 Division of Infectious Diseases, Department of Medicine, New Jersey Medical School, Rutgers, The State University of New Jersey, Newark, New Jersey, United States of America; 6 Cellular and Molecular Immunology Laboratory, Núcleo de Doenças Infecciosas, UFES, Vitória, Brazil; University of California, Davis, United States of America

## Abstract

**Rationale:**

The degree to which tuberculosis (TB) is transmitted between persons is variable. Identifying the factors that contribute to transmission could provide new opportunities for TB control. Transmission is influenced by host, bacterial and environmental factors. However, distinguishing their individual effects is problematic because measures of disease severity are tightly correlated, and assessing the virulence of *Mycobacterium tuberculosis* isolates is complicated by epidemiological and clinical confounders.

**Objectives:**

To overcome these problems, we investigated factors potentially associated with TB transmission within households.

**Methods:**

We evaluated patients with smear-positive (≥2+), pulmonary TB and classified *M. tuberculosis* strains into single nucleotide polymorphism genetic cluster groups (SCG). We recorded index case, household contact, and environmental characteristics and tested contacts with tuberculin skin test (TST) and interferon-gamma release assay. Households were classified as high (≥70% of contacts with TST≥10 mm) and low (≤40%) transmission. We used logistic regression to determine independent predictors.

**Result:**

From March 2008 to June 2012, we screened 293 TB patients to enroll 124 index cases and their 731 contacts. There were 23 low and 73 high transmission households. Index case factors associated with high transmission were severity of cough as measured by a visual analog cough scale (VACS) and the Leicester Cough Questionnaire (LCQ), and cavitation on chest radiograph. SCG 3b strains tended to be more prevalent in low (27.3%) than in high (12.5%) transmission households (p = 0.11). In adjusted models, only VACS (p<0.001) remained significant. SCG was associated with bilateral disease on chest radiograph (p = 0.002) and marginally associated with LCQ sores (p = 0.058), with group 3b patients having weaker cough.

**Conclusions:**

We found differential transmission among otherwise clinically similar patients with advanced TB disease. We propose that distinct strains may cause differing patterns of cough strength and cavitation in the host leading to diverging infectiousness. Larger studies are needed to verify this hypothesis.

## Introduction

Tuberculosis (TB) remains one of the most important threats to global health. Although TB is the prototypic disease with airborne transmission [1], there is both experimental [2], [3] and epidemiologic [4], [5], [6], [7], [8], [9] evidence of marked variability in transmission of *M. tuberculosis* (MTB) from patients with pulmonary TB. The reasons underlying this variability remain poorly understood in part because of the inherent complexity of studying a biological phenomenon resulting from a complex web of interactions between two human hosts–the source case and the exposed contact–and the infecting pathogen within an array of environments. Yet, these differences have important implications for vaccine development and global TB control.

Most studies of transmission and disease in household contacts have focused on host and environmental factors [7], [10]. These studies fail to explain differences in epidemiologic behavior of clinical isolates of MTB reported in outbreaks. In fact, there is a growing body of experimental evidence suggesting that MTB strains have variable degrees of virulence [11], [12], [13], [14], [15], [16], [17], [18], [19]. Although a great deal is known about the genome and proteome of MTB, the genetic basis for most of the cited differences in strain behavior is unknown in most cases [20]. Despite this growing body of literature, there have been no systematic studies designed to investigate the role of strain difference as a determinant of the critical epidemiologic and clinical parameters such as transmissibility, resistance to infection and progression to disease. Importantly, most of the cited studies investigating the virulence potential of MTB isolates have used banked clinical isolates with limited knowledge about the epidemiologic and clinical conditions present at the time the isolates were collected [11], [12], [13], [14], [15], [16], [17], [18], [19], [20].

We conducted a household contact study in Vitória, Brazil to test the hypothesis that genetic strain variation in MTB was a determinant of the extent of transmission of infection in household contacts of smear-positive cases of pulmonary TB. We sought to determine whether some clinical strains of MTB were more transmissible than others, while controlling for other host and environmental factors involved in transmission.

## Methods

### Ethics Statement

The study was approved by the Institutional Review Boards of Boston University Medical Campus and New Jersey Medical School – Rutgers University (formerly University of Medicine and Dentistry of New Jersey), the Comitê de Ética em Pesquisa do Centro de Ciências da Saúde - Universidade Federal do Espírito Santo and the Comissão Nacional de Ética em Pesquisa (CONEP). We obtained written informed consent and assent in Portuguese in accordance with age-specific ethical guidelines of participating institutions.

### Study population

This study was conducted at the The Núcleo de Doenças Infecciosas (NDI) located in Vitória, the capital city of the State of Espírito Santo, Brazil. The NDI has organized a network of five laboratories in the metropolitan region of Vitória that serve 16 TB clinics distributed in the municipalities of Vitória, Cariacica, Serra and Vila Velha. With approximately 1,400 cases/year, the annual TB incidence in Espírito Santo is 38/100,000 inhabitants. The prevalence of HIV infection in Espírito Santo is <1% in the general population, and 7% in TB cases [21].

All consecutive pulmonary TB patients identified through the NDI clinic network were eligible to participate in this study, provided they fulfilled the following inclusion criteria: 1) age ≥18 years with cough ≥3 weeks; 2) new TB episode with ≥1 sputum specimen with acid-fast bacilli (AFB) ≥2+ with subsequent MTB growth in culture; and 3) living with ≥3 household contacts. We excluded index cases who were HIV-infected (or refused HIV testing), had a history of TB treatment, or who were too ill to consent, unable to understand, or to comply with the study protocol. To minimize differences in exposure time between study households, index TB cases were screened and enrolled within the first 2 weeks after they first presented to the municipal TB clinic. A household contact was defined as an individual of any age fulfilling at least one of the following criteria of close contact with the index TB cases for ≥3 months before enrollment: 1) sleeping under the same roof ≥5 days/week; 2) sharing meals ≥5 days/week; 3) watching TV nights or weekends, and; 4) other significant contact (85% of these visited the household >18 days/month).

### Measurements

#### TB cases

We collected demographic and clinical information about TB patients and recorded their cough severity using two measurements: i) a self-reported visual analog cough scale (VACS) [22], and ii) the Leicester Cough Questionnaire (LCQ) [23], [24]. The LCQ is a 19-item, patient-derived questionnaire that measures the physical, psychological and social effects of chronic cough in adults; the final score (range 3–21) is inversely related to cough severity (e.g. higher scores indicate weaker cough). Because of the inherent complexity of the LCQ, study staff administered the LCQ only to TB patients deemed reliable historians (over 95%). We obtained up to three sputa specimens for AFB smear microscopy (auramine O fluorescent stain) [25] and culture (Ogawa-Kudoh method). MTB isolates underwent single nucleotide polymorphism (SNP) analysis as described previously [26] and were categorized into one of nine SNP genetic cluster groups and subgroups (SCG), as described previously [27]. The radiological extent of lung disease was graded on a four-category ordinal scale (normal, minimal, moderate and far-advanced) by an experienced physician [28]. All patients were offered standard TB treatment according to Brazilian guidelines [29], [30]. Study staff visited the participants’ dwellings to verify the identity of each contact, measure individual contact time, and to perform an environmental evaluation (crowding and ventilation).

#### Household contacts

We followed recommendations of the Brazilian TB Control Program for household contact investigations [29], [30]. We recorded demographic and clinical characteristics of contacts, and evaluated them for MTB infection with both TST and IGRA (Quantiferon Gold-In-Tube, Quiagen). To ensure accurate TST readings, only staff trained by the National TB Program were responsible for TST application, and we completed inter- and intra-reader evaluations (kappa >90%) prior to opening study enrollment. Briefly, we placed two units of R23 (SSI, Denmark) provided by the Brazilian Ministry of Health on the forearm of contacts using the Mantoux method; the diameter of induration was measured in millimeters between 72 and 96 hours. Household contacts with a TST<10 mm at baseline were re-tested after 8–12 weeks to identify TST conversion, using two different criteria for conversion: *Criterion 1 (Brazilian Guidelines)*: 1^st^ TST<10 mm, 2^nd^ TST≥10 mm, and difference between 1^st^ and 2^nd^ TST≥10 mm. *Criterion 2:* 1^st^ TST<5 mm, 2^nd^ TST≥10 mm, and difference between 1^st^ and 2^nd^ TST≥6 mm [31]. At 8–12 weeks, blood for IGRA testing was obtained before TST placement to reduce TST-induced IGRA boosting [32]. Following Brazilian recommendations [29], we referred contacts with TST≥10 mm and secondary TB suspects for evaluation and treatment by the corresponding Municipal TB Clinic.

### Household transmission categories

We categorized households into MTB transmission categories according to the percentage of contacts in each household that had a TST≥10 mm by 8–12 weeks: low (LT) transmission (≤40%) or high (HT) transmission (≥70%). This definition includes contacts with TST≥10 mm at either baseline or week 8–12, providing a more complete measure of the total infection burden for each household. Originally, 25% and 75% cutoffs for LT and HT were planned but relaxed to 40% and 70%, respectively, based solely on increasing the numbers in the two categories; no other factors were examined when the cutoffs were revised. A group classified as intermediate transmission (IT) households (41–69% contacts with TST≥10 mm) were also enrolled to serve as controls. Contacts with a history of previous TB disease (N = 13) and with missing results on 1^st^ or 2^nd^ TST (N = 44) were retained in the general analysis but did not contribute to the determination of the transmission category; their inclusion or exclusion had little effect on the category (data not shown). Two contacts were diagnosed with co-prevalent TB disease and based on symptom history were determined to have been infected by the index case and not the reverse; these two contacts were considered TST-positive for purposes of the determination of the transmission category.

### Statistical methods

Because this study used a household contact design, characteristics are at both a household level (including data about the index case) and at an individual contact level, nested within households. Households were classified into three phenotypes, i.e., low, intermediate, and high transmission. Comparisons were made between LT and HT households, and also across the three phenotypes. Unadjusted associations of household level data to transmission phenotype assumed independence across households. Logistic regression was used to obtain odds ratios of household level characteristics, but in the case of sparse data, exact logistic regression and corresponding odds ratios and tests were substituted [33]. Characteristics from individual contacts within a household were potentially correlated and, therefore, were evaluated using a generalized estimating equation (GEE) approach with an independent working correlation structure. We assumed a binomial distribution with a logit link when comparing LT and HT households and a multinomial distribution with a cumulative logit link when comparing LT, IT, and HT households. Multiple regression modeling focused on comparing LT vs. HT households and used the GEE approach to account for within household correlation. An adhoc approach was used to determine characteristics for the final multiple regression model. An automatic stepwise selection procedure was used to select household level characteristics, and then individual contact level covariates were candidate for addition to the model in a manual stepwise fashion. SCG and contact age were then added to the model. Interactions of SCG with measures of disease severity used simplified models because of estimation problems. Covariates of questionable reliability were excluded from consideration. Because of their strong negative correlation (Pearson correlation −0.72*) VACS and LCQ were not candidates for inclusion in a single multivariable model. Direct comparison of VACS and LCQ showed that VACS was a superior predictor of transmission category based on both Akaike Information criterion (AIC) and likelihood score statistic. Moreover, LCQ is time consuming and difficult to administer and score. Therefore, multivariable analyses only considered inclusion of VACS. All analyses were conducted in SAS 9.2 and testing is two-sided.

## Results

**Figure 1:**
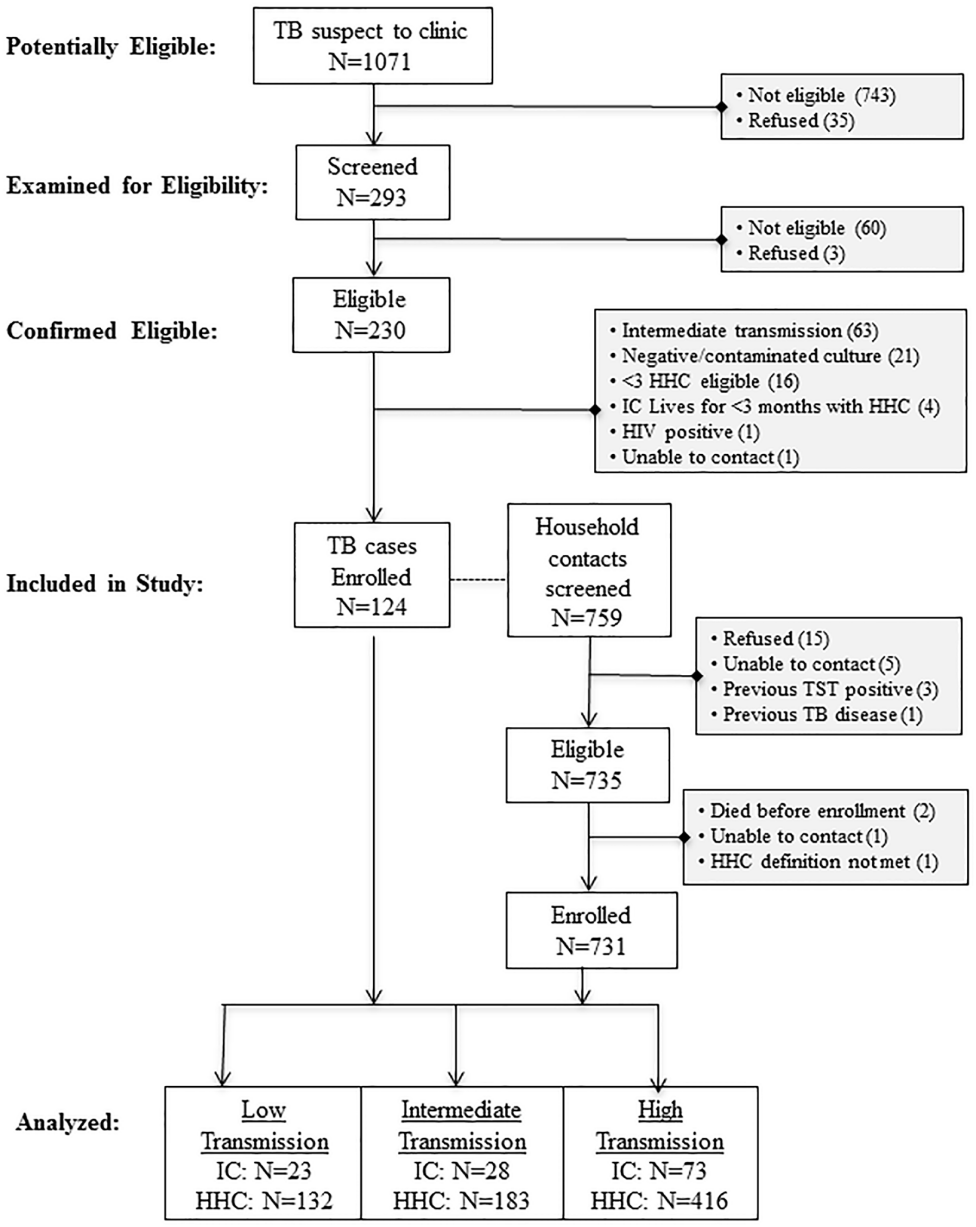
Study profile.

Between February 2008 and June 2012, we identified 1,071 potentially eligible TB patients, 743 were determined ineligible and not referred to the study, 35 refused study participation, and 293 underwent screening; of these, 230 families were confirmed eligible, but 106 were not enrolled (Figure 1). This analysis includes 124 index cases and their 731 household contacts: 23 LT families (132 household contacts), 73 HT families (416 contacts), and 28 IT families (183 contacts) that were selected for enrollment as control households.

### Index TB patients and dwellings

The median age of TB cases was 36 years (range 25, 46), 64% were male, and most had advanced TB disease as measured by duration of cough (median 12 weeks), sputum AFB smear (82% AFB 3+), or extent of lung disease on chest radiograph (Table 1). The severity of cough (as measured by VACS and LCQ) and the presence of cavitation on chest radiograph in index TB cases increased as the proportion of household members with MTB infection increased. There were no differences in socio-economic variables (marital status, highest education level, occupation and average salary) between household transmission categories, except that families living in HT households were more likely to rent (P = 0.012). When compared to LT households, contacts from IT and HT families were more likely to self-report close contact with another person with TB other than the index case (P = 0.02). Study dwellings were similar in terms of building materials, sanitation, size, crowding, ventilation, and other examined factors (Table S1).

**Table 1:** Characteristics of index tuberculosis cases, household contacts and study dwellings at baseline by *Mycobacterium tuberculosis* transmission category.

Characteristic^1^		Final Household transmission category				P value 2 (Multinomial)	P value 2 (LT vs. HT)
		Total	Low (LT)	Intermediate	High (HT)		
***Index Cases***		*N = 124*	*N = 23*	*N = 28*	*N = 73*		
Age (years)		36 [25], [46]	36 [28], [49]	38 [29], [46]	33 [22], [46]	0.34K	0.47W
Sex						0.77C	0.58C
	Male	80 (64.5)	14 (60.9)	17 (60.7)	49 (67.1)		
	Female	44 (35.5)	9 (39.1)	11 (39.3)	24 (32.9)		
Cough visual analog scale		7 [4], [9]	5 [3], [6]	6 [4], [9]	8 [6], [9]	0.003K	0.002W
Leicester cough questionnaire		11 [10], [14]	13 [11], [17]	12 [10], [16]	11 [9], [13]	0.007K	0.007W
Weeks sick prior to enrollment		12 [4], [20]	8 [4], [24]	8 [4], [18]	12 [8], [24]	0.26K	0.57W
Housing						0.017T	0.11E
	Owned	107 (86.3)	22 (95.7)	27 (96.4)	58 (79.5)		
	Rented	17 (13.7)	1 (4.3)	1 (3.6)	15 (20.5)		
Sputum AFB smear (highest grade)						0.49C	0.30C
	2+	22 (17.7)	6 (26.1)	4 (14.3)	12 (16.4)		
	3+	102 (82.3)	17 (73.9)	24 (85.7)	61 (83.6)		
Extent of lung disease on chest radiograph						0.20J	0.24T
	Normal	2 (1.7)	1 (4.3)	1 (3.7)	0 (0.0)		
	Minimal	7 (5.8)	1 (4.3)	2 (7.4)	4 (5.6)		
	Moderate	63 (52.1)	13 (56.5)	15 (55.6)	35 (49.3)		
	Far advanced	49 (40.5)	8 (34.8)	9 (33.3)	32 (45.1)		
Cavitations						0.012T	0.023T
	Absent	35 (28.9)	10 (43.5)	11 (40.7)	14 (19.7)		
	Present	86 (71.1)	13 (56.5)	16 (59.3)	57 (80.3)		
SNP cluster group						0.38T	0.46E
	3b	20 (16.8)	6 (27.3)	5 (20.0)	9 (12.5)		
	4	2 (1.7)	0 (0.0)	1 (4.0)	1 (1.4)		
	5	88 (73.9)	15 (68.2)	18 (72.0)	55 (76.4)		
	6a	8 (6.7)	1 (4.5)	1 (4.0)	6 (8.3)		
	5 or 6a^3^	1 (0.8)	0 (0.0)	0 (0.0)	1 (1.4)		
Number of contacts enrolled in study		5 [4], [7]	5 [4], [7]	6 [5,7.5]	5 [4], [7]	0.10K	0.98W
***Household Contacts***		*N = 731*	*N = 132*	*N = 183*	*N = 416*		
Age (years)		20 [9], [39]	18.5 [8.5,38.5]	25 [12], [41]	19 [9], [37]	0.65S	0.80S
Sex						0.85S	0.40S
	Male	325 (44.5)	63 (47.7)	76 (41.5)	186 (44.7)		
	Female	406 (55.5)	69 (52.3)	107 (58.5)	230 (55.3)		
BCG scar						0.72S	0.86S
	Present	563 (77.0)	107 (81.1)	130 (71.0)	326 (78.4)		
	Absent	137 (18.7)	21 (15.9)	40 (21.9)	76 (18.3)		
	Uncertain	31 (4.2)	4 (3.0)	13 (7.1)	14 (3.4)		
Close contact with person with TB other than index case						0.020S	<0.001S
	Yes	159 (21.8)	7 (5.3)	46 (25.1)	106 (25.5)		
	No	570 (78.1)	124 (93.9)	137 (74.9)	309 (74.5)		
TST and IGRA status, n/N (%)							
1^st^ TST≥10 mm (at baseline)		407/710 (57.3)	17/132 (12.9)	78/180 (43.3)	312/398 (78.4)		
1^st^ or 2^nd^ TST≥10 mm (at 8–12 weeks)		488/691 (71.7)	27/127 (21.3)	103/174 (59.2)	358/380 (94.2)	-	-
	≤5 years	48/85 (56.5)	0/20 (0.0)	9/23 (39.1)	39/42 (92.9)	-	-
	6–15 years	129/190 (67.9)	3/36 (8.3)	14/36 (38.9)	112/118 (94.9)	-	-
	≥16 years	311/406 (76.6)	24/71 (33.8)	80/115 (69.6)	207/220 (94.1)	-	-
Median diameter (mm)							
	All	14 [7], [17]	14 [7], [17]	10 [2], [15]	16 [13], [18]	-	-
	Among TST≥10 mm (only)	16 [13], [19]	14 [12], [17]	15 [11], [19]	16 [14], [19]	<0.001S	<0.001S
Positive IGRA (at 8–12 weeks)		322/566 (56.9)	18/101 (17.8)	62/149 (41.6)	242/316 (76.6)	-	-
	≤5 years	19/47 (40.4)	0/10 (0.0)	3/13 (23.1)	16/24 (66.7)	-	-
	6–15 years	95/160 (59.4)	5/30 (16.7)	9/30 (30.0)	81/100 (81.0)	-	-
	≥16 years	208/359 (57.9)	13/61 (21.3)	50/106 (47.2)	145/192 (75.5)	-	-

1 n (%) or median [IQR], unless otherwise specified.

2 C = Chi-squared, E = Exact, K = Kruskal-Wallis, J = Jonckheere-Trepstra, T = Cochran-Armitage Trend, M = McNemar’s Test, S = Score test from generalized estimating equation (GEE) logistic model; W = Wilcoxon rank sum test.

3 The SNP cluster group for one index case could not be distinguished between 5 or 6a. This participant’s result is shown in the table but is excluded when performing statistical tests.

Missing data: Leicester cough questionnaire (6), cough visual analog scale (6), chest radiograph (3), contact with other TB person than index case (1 missing, 1 don’t know), 1^st^ TST (21), 1^st^ or 2^nd^ TST (40), IGRA (161 missing, 4 indeterminate), SNP (5).

Infection status of contacts is shown at baseline and at study conclusion (8 weeks).

### Genotypic strain analysis

**Figure 2:**
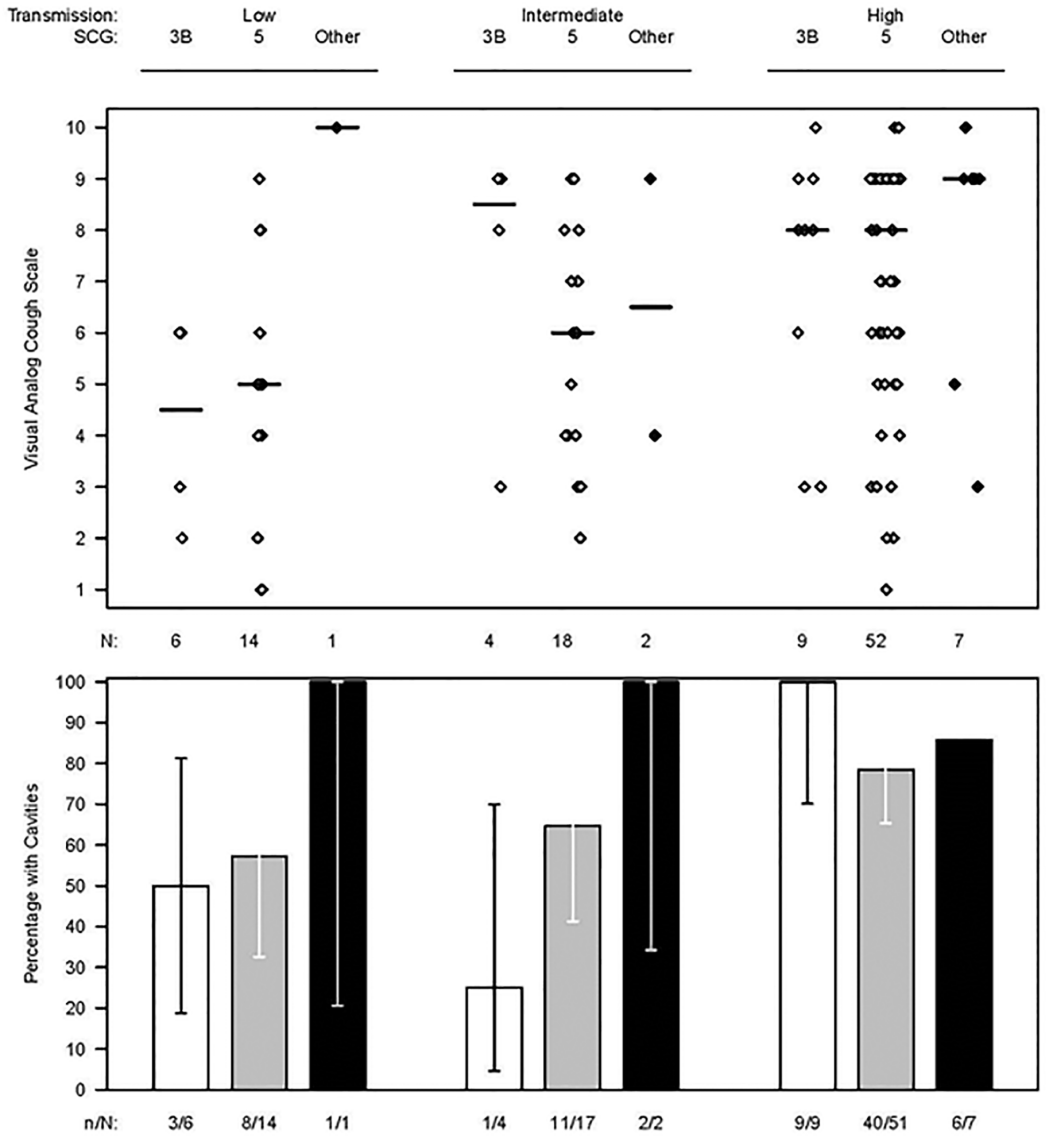
Cough severity (as measured by visual analog cough scale) in index TB case by household transmission category, single nucleotide polymorphism genetic cluster group (SCG), and cavitations on chest radiograph (N = 111).

The two most prevalent strains among TB patients were group 5 (73.9%) and group 3b (16.8%) and their distribution across the three household transmission categories differed qualitatively, although not statistically significantly (omnibus P = 0.38) (Table 1). Group 3b strains were more prevalent in LT (27.3%) and IT (20.0%) households compared to HT (12.5%) households; conversely, group 5 strains were most prevalent in HT (76.4%) households compared to IT (68.2%) and LT (72.0%) households. The median [interquartile range (IQR)] LCQ score was 12 (7, 21) for patients infected with SCG 3b strains, 11 (6, 21) for SCG 5, and 10 (5, 16) for other SCG groups combined (p = 0.056), trending toward less coughing in patients infected with a SCG 3b isolate. Similarly, the median (IQR) VACS was 6 (0, 10) for SCG 3b TB patients (N = 19), 7 (0, 10) for SCG 5 (N = 83), and 9 (3, 10) for all other groups (N = 11) combined (p = 0.11), indicating patients infected with a SCG 3b strain had a trend toward a weaker cough (Figure 2). Patients with SCG 3b isolates were less likely to have bilateral disease (15.0%), vs. SCG 5 (51.2%) and other SCG (72.7%) (p = 0.002), and less likely to have infiltrates in the upper lung: SCG 3b (80.0%), 5 (95.3%), and 4 or 6a (100.0%), with the differences being marginally significant (p = 0.060). Similarly, cavitations were present in SCG 3b (65.0%), 5 (71.8%), and 4 or 6a (90.0%) but these differences were not statistically significant (p = 0.39).

### Household contacts

This analysis includes a total of 731 household contacts that fulfilled the study definition of close and prolonged contact time with the index case (categorized by the first definition met): 63% slept under the same roof ≥5 days/week, 12% watched TV on nights/weekends, 11% shared meals ≥5 days/week and 14% had other significant contact (85% visited the household >18 days/month). Most (90%) households were evaluated within ±14 days of the index TB cases starting anti-tuberculous treatment. Household contacts were young (median age 20 years), but covering a broad age range (3 months to 87 years); 56.3% were female; 77.0% had a BCG vaccination scar; 99% had no prior diagnosis of MTB and 98% reported no HIV-infection (Table 1). Enrolled contacts spent considerable time in close contact with their family member with TB. Contacts were comparable across transmission categories in terms of socio-economic condition (marital status, education level, occupation and salary), family composition (i.e. no new contacts left the household or died in 3 months prior to study inception), alcohol and tobacco consumption, and history of co-morbidities.

At baseline, TST was positive in 57.3% of contacts and the distribution varied according to transmission category (by design) and contact age (Table 1). Whereas the percentage of contacts with TST≥10 mm living in LT and IT households increased with age, it was similar across age strata in HT households. Overall, the frequency of IGRA positivity at 8–12 weeks was approximately 15% lower than TST but showed a similar age-dependent increase in prevalence of infection. The median TST diameter in contacts with TST≥10 mm (1^st^ or 2^nd^ TST) increased in step with household transmission categories (P<0.001).

### Household transmission categories

Transmission categories of households changed considerably between baseline and week 8–12 as a result of TST conversion (Tables 2 and 3). At baseline, 41, 30 and 53 of the sampled households were LT, IT and HT, respectively; by 8–12 weeks, 43% of LT and IT household progressed and only 23 remained as LT households (Table 2). The use of two different criteria to define TST conversion identified the same number of contacts with newly acquired infection, however the same contacts were not identified by both criteria (Table 3); of the 56 contacts that underwent TST conversion by either criterion, 44 (79%) fulfilled both TST conversion criteria. Most TST conversions occurred in households initially classified as low or intermediate households, as the proportion of contacts “at risk” of TST conversion living in HT households was lower by definition. Adolescent contacts were more likely to convert their TST than adults (conversion criterion 1, P = 0.025).

**Table 2:** Distribution of households according to study *M. tuberculosis* transmission categories by study time point.

Study time point		Final transmission category (2^nd^ TST)		
		n (row %)		
Initial transmission category (1^st^ TST)		Low	Intermediate	High
n				
**Low**	41	23 (56)	11 (27)	7 (17)
**Intermediate**	30	NA	17 (57)	13 (43)
**High**	53	NA	NA	53 (100)
	Totals	23	28	73

NA = Not applicable.

**Table 3:** Household contacts with tuberculin skin test (TST) conversion from entry to week 8–12 by initial household transmission category and age of contact.

TST conversion characteristic	Initial (1^nd^ TST) household transmission category				P* value
	No. with TST conversion/No. at risk (%)				
	All	Low	Intermediate	High	
**Criterion 1** 1					
All	50/271 (18.4)	36/189 (19.0)	13/72 (18.1)	1/10 (9.1)	0.67
≤5 years	5/43 (11.6)	4/32 (12.5)	1/11 (9.1)	0/0 (0.0)	0.75
6–15 years	23/86 (26.7)	17/63 (27.0)	6/20 (30.0)	0/3 (0.0)	0.87
≥16 years	22/142 (15.5)	15/94 (16.0)	6/41 (14.6)	1/7 (12.5)	0.77
**Criterion 2** 2					
All	50/221 (22.6)	36/159 (22.6)	13/53 (24.5)	1/9 (11.1)	0.93
≤5 years	5/42 (11.9)	4/31 (12.9)	1/11 (9.1)	0/0 (0.0)	0.72
6–15 years	23/76 (30.3)	18/57 (31.6)	5/16 (31.3)	0/3 (0.0)	0.87
≥16 years	22/103 (21.4)	14/61 (19.7)	7/26 (26.9)	1/6 (16.7)	0.64

n/N are number of converters divided by the number at risk as defined by the various criteria below.

1 TST conversion Criterion 1 (Brazilian guidelines): 1^st^ TST<10 mm; 2^nd^ TST≥10 mm; difference ≥10 mm. Does not include contacts with 1^st^ TST≥10 mm (407, 56%), and those with missing 1^st^ (21, 3%) or 2^nd^ TST (31, 4%) because they are not considered “at risk” by this criterion.

2 TST conversion Criterion 2: 1^st^ TST<5 mm; 2^nd^ TST≥10 mm; difference ≥6 mm. Does not include contacts with 1^st^ TST≥5 mm (458, 65%), and those with missing 1^st^ TST (21, 3%) or 2^nd^ TST (31, 4%) because they are not considered “at risk” by this criterion.

*Score test from generalized estimating equation (GEE) estimation approach to logistic regression.

Only contacts “at risk” of TST conversion are included for each criterion.

### Factors associated with high MTB transmission

We analyzed index TB patient, household contact and environmental factors associated with HT households (Table 4). In unadjusted analyses, factors associated with HT were the presence of cavitation in chest radiograph [Odds Ratio (OR) 3.13, 95% confidence interval (CI): 1.13, 8.69; P = 0.028] and stronger cough as measured by VACS (OR 1.36 per unit increase, 95% CI: 1.13, 1.67; P = 0.001) or LCQ (OR 1.24 per unit decrease, 95% CI: 1.07, 1.45; P = 0.005) in index TB cases. A group 5 SCG MTB isolate had more than twice the estimated odds of being associated with HT compared to group 3b, although it was not statistically significant (OR 2.44, 95% CI: 0.72, 7.93; P = 0.14). In a logistic regression model adjusted for other covariates with a GEE approach for estimation, the only variable that remained independently associated with HT was severity of cough in the index TB case (VACS adjusted OR per unit increase 1.53, 95% CI: 1.26, 1.86; P<0.001). The presence of cavitation on chest radiograph trended toward significance (P = 0.078), age of the contact (P = 0.17) and SCG (p = 0.40) were not significantly associated with a HT household. We found no evidence of interaction between SCG and either cavitation (p = 0.50) or VACS (p = 0.49) but were underpowered for such comparisons and in fact had estimation problems.

**Table 4:** Unadjusted and Adjusted Odds Ratios of Index TB Case, Household Contact and Environmental Factors for Predicting High *Mycobacterium tuberculosis* Transmission Households.

Characteristic	Household Transmission Category		Unadjusted^1^ OR	P-value*	P-value+	Adjusted^2^ OR	P-value*	P-value+
	n (column %)		[95% CI]			[95% CI]		
	Low	High						
***Index cases***	*N = 23*	*N = 73*						
Age (per 10-year increment)	23	73	0.90 [0.62, 1.32]	0.59	0.59			
Cough measurement at baseline								
VACS (per unit increase)	21	70	1.36 [1.13, 1.67]	0.002	0.001	1.53 [1.27, 1.86]		<0.001
LCQ (per unit decrease)	21	70	1.24 [1.07, 1.45]	0.007	0.005			
Chest radiograph					0.64E			
Normal/Minimal	2 (8.3)	4 (5.6)	1 (ref)					
Moderate	13 (56.5)	35 (49.3)	1.34 [0.11, 10.7]E	1.00E				
Far advanced	8 (34.8)	32 (45.1)	2.00 [0.25, 12.33]E	0.78E				
Cavitations					0.028			0.078
Absent	10 (43.5)	14 (19.7)	1 (ref)			1 (ref)		
Present	13 (56.5)	57 (80.3)	3.13 [1.13, 8.69]	0.027		3.50 [0.97, 12.58]		
Sputum AFB smear					0.31			
2+	6 (26.1)	12 (16.4)	1 (ref)					
3+	17 (73.9)	61 (83.6)	1.79 [0.56, 5.38]	0.30				
SNP cluster group					0.21			0.40
3b	6 (27.3)	9 (12.7)	1 (ref)			1 (ref)		
5	15 (68.2)	55 (77.5)	2.44 [0.72, 7.93]	0.14		2.93 [0.74, 11.64]	0.13	
Other 3	1 (4.5)	7 (9.9)	5.33 [0.70, 112.44]	0.16		2.45 [0.15, 39.91]	0.53	
***Household contacts***	*N = 132*	*N = 416*						
Age (years)					0.47			0.17
≤5	21 (15.9)	48 (11.5)	1 (ref)			1 (ref)		
6–15	37 (28.0)	127 (30.5)	1.50 [0.78, 2.89]	0.22		1.63 [0.77, 3.46]	0.23	
≥16	74 (56.1)	241 (57.9)	1.42 [0.84, 2.43]	0.19		1.92 [1.08, 3.39]	0.026	
BCG scar					0.85			
Absent	21 (15.9)	76 (18.3)	1 (ref)					
Present	107 (81.1)	326 (78.4)	0.84 [0.43, 1.63]	0.43				
Uncertain	4 (3.0)	14 (3.4)	0.97 [0.23, 3.99]	0.23				
Average exposure time to index case last 3 months (hours/day)					0.41			
<7	38 (28.8)	152 (36.5)	1 (ref)					
7–12	45 (34.1)	98 (23.6)	0.54 [0.22, 1.33]	0.18				
13–18	31 (23.5)	123 (29.6)	0.99 [0.34, 2.90]	0.99				
>18	18 (13.6)	43 (10.3)	0.60 [0.20, 1.79]	0.36				
***Environment***								
Sleeping arrangement with index case					0.51			
Same house								
Same room, same bed	16 (12.1)	70 (16.8)	1 (ref)					
Same room, different bed	14 (10.6)	30 (7.2)	0.49 [0.18, 1.35]	0.17				
Different room	61 (46.2)	178 (42.8)	0.67 [0.32, 1.40]	0.28				
Different house	41 (31.1)	138 (33.2)	0.77 [0.28, 2.11]	0.61				

1 Household level data, except chest radiograph (see footnote E below), are modeled using logistic regression. Contact level data are modeled by using generalized estimating equations (GEE) approach to estimation for logistic regression with an independent working correlation matrix with standard errors obtained from a sandwich estimate of the variance.

2 Fit using a GEE approach to estimation for logistic regression with an independent working correlation matrix adjusted for all other covariates listed. Standard errors are from a sandwich estimator of the variance.

3 One participant whose SCG could not be distinguished between 5 or 6a was excluded.

*Wald (pairwise vs. reference) P-value.

+ Global (omnibus) score P-value.

E = denotes exact Odds Ratios and P-values using an exact test of the parameters from exact logistic regression.

VACS = Visual analog cough scale; LCQ = Leicester cough questionnaire.

BCG = Bacille Calmette Guérin vaccine; TST = Tuberculin skin test; OR = Odds ratio; CI = Confidence Interval.

## Discussion

In this household contact study of smear-positive, culture confirmed pulmonary TB patients in Brazil, we systematically identified households with low and high transmission of MTB during a four-year period. Our study design and use of both TST and IGRA to measure prevalent and incident infection in exposed contacts showed heterogeneity of MTB transmission within households. We also found that cough severity in the index case is significantly associated with high transmission and that the presence of cavitation on chest radiograph is also associated, albeit marginally, with increased transmission. Our findings indicate that the infecting strain may result in distinct patterns of pathophysiology of disease in index cases leading to differences in their infectiousness.

The importance of cough frequency as a surrogate for TB infectiousness is well recognized [34], [35], [36], [37]. Overnight cough frequency has been associated with the proportion of contacts with a positive TST [35]. However, the objective evaluation of cough, an inherently subjective symptom, is challenging and we are unaware of previously published data relating cough strength or other measures of cough physiology with MTB transmission. Also, hosts may be more infectious during a specific time of day so there is a need for an objective and quantitative measure of infectiousness [35], [38]. Similarly, a hallmark of TB disease is the formation of lung cavitation, the result of liquefaction of tissue after the initial granulomatous response [39]. With progression of disease, cavitation becomes more common, occurring in 45–91% of cases [40]; when present, cavities are associated with a higher bacillary load and increased transmission [35], [39], [41] when compared to patients with nodular lesions in the lung parenchyma [42], [43], [44]. However, measures of pulmonary TB disease severity such as cough strength, cavitation, and bacterial load in sputum are tightly correlated and distinguishing their individual effects on MTB transmission can be challenging. We addressed this in our study design by using two separate instruments to measure cough (VACS and LCQ), recording the presence of cavitations on chest radiograph and restricting enrollment to smear positive (≥2+), culture-confirmed TB patients in an effort to maximize and homogenize already proven exposure across households. Whereas the VACS and LCQ have been validated in other conditions with chronic cough (e.g. chronic obstructive pulmonary disease, asthma), to our knowledge this is the first study in which they have been applied to patients with pulmonary TB.

There is accumulating evidence that strain diversity among MTB may result in clinically meaningful differences in disease outcome. Traditionally, MTB strain virulence has been defined based on phenotypic properties in varying conditions such as increased production of pro-inflammatory cytokines in the source or contact cases, growth characteristics in liquid cultures, macrophages, and THP1 cells, and growth kinetics in animal experiments [16], [17], [18]. Although some of the in-vitro data show conflicting results [45], [46], current thinking is that virulent strains subvert the immune system by causing a reduced inflammatory response, which in turn results in impaired bacterial control by the host, more rapidly progressive disease, and enhanced transmission. Strain virulence also can be inferred from the capacity of MTB clones to spread in human populations, as measured by the identification of strain clusters using genotyping techniques [11], [15], [19], [47]. However, it remains unclear whether there is a microbial basis to explain why some clinical isolates cause widespread disease and other closely related strains remain limited in spread [46], [48], [49]. Our study suggests that SCG 3b strains are associated with diminished transmission but that this may be due to weaker cough and decreased cavity formation, opening the possibility of strain-dependent variable lung parenchymal pathology and/or inflammation in the index TB patient. In fact, the hallmark of the local immune response in pulmonary TB is an unregulated activation of cells and expression of pro-inflammatory and anti-inflammatory cytokines and chemokines [50]. SCG 3b roughly corresponds to the H spoligotype; SCG5, the other SCG which was commonly observed in our index cases, roughly corresponds to the LAM spoligotype. Both LAM and H spoligotypes are members of the Euro-American LSP/SNP based lineage [51]. This lineage is distinct from the East Asian tuberculosis lineages that includes Beijing type strains, which have been hypothesized to have increased virulence. Both SCG3b and SCG5 strains are expected to differ by a relatively small number of mutations because they are situated quite close to each other on SNP generated phylogenies, compared to more distantly related lineages such as the Beijing strains [26]. This suggests that only a small number of mutations may be responsible for the change in transmissibility observed in SC3b strains. These mutations may be discoverable in future whole genome sequencing studies.

One important limitation has been that most studies examining the virulence potential of clustered MTB isolates have used banked isolates with limited knowledge about the epidemiologic and clinical conditions present at the time the strains were cultured, and thus fail to consider potential confounders related to characteristics of the index case (e.g. cough severity, bacterial load, cavitations), environment (e.g. ventilation, crowding) or contact (e.g. age, BCG and proximity of contact) [10]. Household contact studies such as this one are a classic, well-accepted design to study MTB transmission dynamics in a well-characterized group of individuals with a defined infectious challenge, while controlling for host, environmental and bacterial confounders. However, contacts that are already infected at the time of initial ascertainment may have been infected by the source case or, in an intermediate prevalence country such as Brazil, infected in the community [52], [53]. As observed in our study, this translates into age-dependent differences in prevalence of infection in contacts (i.e. higher frequency of TB exposures in adults as a result of wider social networks).

Our study has limitations. Whereas all patients were sputum smear- and culture-positive on solid media, we did not obtain quantitative culture data, which is known as a stronger surrogate of bacterial load and risk of infection. Between February 2008 and April 2009, we obtained a repeat TST in all contacts with TST <10 mm at study screening, including those living in IT households. As some IT households were excluded at the 1^st^ TST after April 2009, some of which presumably would have been re-classified as HT, we cannot estimate the percentage of households that are HT within the study community. IT households are also underrepresented. However, because the initially HT group of households was large, the inclusion of additional households through conversion between the two TST applications into this group are unlikely to have altered the results of this study. Also, whereas our criteria to classify households were operationally driven, the definition of HT was robust in that 77% of HT households had 100% of households with TST≥10 mm.

In conclusion, in this household contact study of culture confirmed, AFB 2+ smear-positive TB patients in a Brazilian urban setting we observed variable MTB transmission within households. Our results provide strong evidence of differential transmission among otherwise clinically similar patients with advanced TB disease. Data presented here also suggest that these differences in infectiousness may be modulated by yet unknown interactions between the host and the infecting strain, resulting in weaker cough and possibly, less formation of cavities. Further studies will be needed to mechanistically advance these novel findings.

## Supporting Information

Table S1 **Additional characteristics of index tuberculosis cases, household contacts, and study dwellings at baseline by** ***Mycobacterium tuberculosis*** **transmission category.** ^1^n (%) or median [IQR]. ^2^K = Kruskal-Wallis, J = Jonckheere-Trepstra, T = Cochran-Armitage Trend, S = Score test from generalized estimating equation (GEE); W = Wilcoxon rank sum test Missing data: BMI (2).
(DOCX)

## References

[1] Roy CJ, Milton DK (2004) Airborne transmission of communicable infection–the elusive pathway. The New England journal of medicine 350: 1710–1712. 15102996 10.1056/NEJMp048051

[2] Escombe AR, Moore DA, Gilman RH, Pan W, Navincopa M, et al (2008) The infectiousness of tuberculosis patients coinfected with HIV. PLoS medicine 5: e188. 18798687 10.1371/journal.pmed.0050188 PMC2535657

[3] Sultan L, Nyka W, Mills C, O’Grady F, Wells W, et al.. (1960) Tuberculosis disseminators: A study of the variability of aerial infectivity of tuberculous patients. The American review of respiratory disease 82: 358–369. PMID: 13835667. PMCID journal - in process. 10.1164/arrd.1960.82.3.358 13835667

[4] Borgdorff M, W,, Nagelkerke NJ, de Haas PE, van Soolingen D (2001) Transmission of Mycobacterium tuberculosis depending on the age and sex of source cases. American journal of epidemiology 154: 934–943. PMID: 11700248. PMCID journal - in process. 10.1093/aje/154.10.934 11700248

[5] Brooks SM, Lassiter NL, Young EC (1973) A pilot study concerning the infection risk of sputum positive tuberculosis patients on chemotherapy. The American review of respiratory disease 108: 799–804. 4741874 10.1164/arrd.1973.108.4.799

[6] Hamburg MA, Frieden TR (1994) Tuberculosis transmission in the 1990s. The New England journal of medicine 330: 1750–1751. PMID: 7910663. PMCID journal - in process. 10.1056/NEJM199406163302410 7910663

[7] Morrison J, Pai M, Hopewell PC (2008) Tuberculosis and latent tuberculosis infection in close contacts of people with pulmonary tuberculosis in low-income and middle-income countries: a systematic review and meta-analysis. The Lancet infectious diseases 8: 359–368. 18450516 10.1016/S1473-3099(08)70071-9

[8] van Geuns HA, Meijer J, Styblo K (1975) Results of contact examination in Rotterdam, 1967–1969. Bulletin of the International Union against Tuberculosis 50: 107–121. 1218286

[9] Verhagen LM, van den Hof S, van Deutekom H, Hermans PW, Kremer K, et al (2011) Mycobacterial factors relevant for transmission of tuberculosis. The Journal of infectious diseases 203: 1249–1255. 21378376 10.1093/infdis/jir013

[10] Guwatudde D, Nakakeeto M, Jones-Lopez EC, Maganda A, Chiunda A, et al (2003) Tuberculosis in household contacts of infectious cases in Kampala, Uganda. American journal of epidemiology 158: 887–898. 14585767 10.1093/aje/kwg227 PMC2869090

[11] Hanekom M, Gey van Pittius NC, McEvoy C, Victor TC, Van Helden PD, et al (2011) Mycobacterium tuberculosis Beijing genotype: a template for success. Tuberculosis 91: 510–523. 21835699 10.1016/j.tube.2011.07.005

[12] Dormans J, Burger M, Aguilar D, Hernandez-Pando R, Kremer K, et al (2004) Correlation of virulence, lung pathology, bacterial load and delayed type hypersensitivity responses after infection with different Mycobacterium tuberculosis genotypes in a BALB/c mouse model. Clinical and experimental immunology 137: 460–468. 15320894 10.1111/j.1365-2249.2004.02551.x PMC1809137

[13] Palanisamy GS, DuTeau N, Eisenach KD, Cave DM, Theus SA, et al (2009) Clinical strains of Mycobacterium tuberculosis display a wide range of virulence in guinea pigs. Tuberculosis 89: 203–209. 19251482 10.1016/j.tube.2009.01.005

[14] Portevin D, Gagneux S, Comas I, Young D (2011) Human macrophage responses to clinical isolates from the Mycobacterium tuberculosis complex discriminate between ancient and modern lineages. PLoS pathogens 7: e1001307. 21408618 10.1371/journal.ppat.1001307 PMC3048359

[15] Hanekom M, van der Spuy GD, Streicher E, Ndabambi SL, McEvoy CR, et al (2007) A recently evolved sublineage of the Mycobacterium tuberculosis Beijing strain family is associated with an increased ability to spread and cause disease. J Clin Microbiol 45: 1483–1490. 17360841 10.1128/JCM.02191-06 PMC1865897

[16] Lopez B, Aguilar D, Orozco H, Burger M, Espitia C, et al (2003) A marked difference in pathogenesis and immune response induced by different Mycobacterium tuberculosis genotypes. Clinical and experimental immunology 133: 30–37. 12823275 10.1046/j.1365-2249.2003.02171.x PMC1808750

[17] Manca C, Reed MB, Freeman S, Mathema B, Kreiswirth B, et al (2004) Differential monocyte activation underlies strain-specific Mycobacterium tuberculosis pathogenesis. Infection and immunity 72: 5511–5514. 15322056 10.1128/IAI.72.9.5511-5514.2004 PMC517425

[18] Theus SA, Cave MD, Eisenach KD (2004) Activated THP-1 cells: an attractive model for the assessment of intracellular growth rates of Mycobacterium tuberculosis isolates. Infection and immunity 72: 1169–1173. 14742569 10.1128/IAI.72.2.1169-1173.2004 PMC321586

[19] Theus SA, Cave MD, Eisenach KD (2005) Intracellular macrophage growth rates and cytokine profiles of Mycobacterium tuberculosis strains with different transmission dynamics. The Journal of infectious diseases 191: 453–460. 15633105 10.1086/425936

[20] Tsenova L, Ellison E, Harbacheuski R, Moreira AL, Kurepina N, et al (2005) Virulence of selected Mycobacterium tuberculosis clinical isolates in the rabbit model of meningitis is dependent on phenolic glycolipid produced by the bacilli. The Journal of infectious diseases 192: 98–106. 15942899 10.1086/430614

[21] Prado TN, Caus AL, Marques M, Maciel EL, Golub JE, et al (2011) Epidemiological profile of adult patients with tuberculosis and AIDS in the state of Espirito Santo, Brazil: cross-referencing tuberculosis and AIDS databases. Jornal brasileiro de pneumologia: publicacao oficial da Sociedade Brasileira de Pneumologia e Tisilogia 37: 93–99. 21390437 10.1590/s1806-37132011000100014 PMC3713784

[22] Raj AA, Birring SS (2007) Clinical assessment of chronic cough severity. Pulmonary pharmacology & therapeutics 20: 334–337. 17113330 10.1016/j.pupt.2006.10.002

[23] Birring SS, Prudon B, Carr AJ, Singh SJ, Morgan MD, et al (2003) Development of a symptom specific health status measure for patients with chronic cough: Leicester Cough Questionnaire (LCQ). Thorax 58: 339–343. 12668799 10.1136/thorax.58.4.339 PMC1746649

[24] Leconte S, Ferrant D, Dory V, Degryse J (2011) Validated methods of cough assessment: a systematic review of the literature. Respiration; international review of thoracic diseases 81: 161–174. 21079381 10.1159/000321231

[25] Organization. WH (1998) Laboratory Services in Tuberculosis Control: Part II Microscopy. WHO/TB/98.258 WHO/TB/98.258.

[26] Filliol I, Motiwala AS, Cavatore M, Qi W, Hazbon MH, et al (2006) Global phylogeny of Mycobacterium tuberculosis based on single nucleotide polymorphism (SNP) analysis: insights into tuberculosis evolution, phylogenetic accuracy of other DNA fingerprinting systems, and recommendations for a minimal standard SNP set. Journal of bacteriology 188: 759–772. 16385065 10.1128/JB.188.2.759-772.2006 PMC1347298

[27] Alland D, Lacher DW, Hazbon MH, Motiwala AS, Qi W, et al (2007) Role of large sequence polymorphisms (LSPs) in generating genomic diversity among clinical isolates of Mycobacterium tuberculosis and the utility of LSPs in phylogenetic analysis. Journal of clinical microbiology 45: 39–46. 17079498 10.1128/JCM.02483-05 PMC1828963

[28] Falk A OCJ, Pratt C. (1969) Classification of pulmonary tuberculosis. In: Diagnostic standards and classification of tuberculosis.. New York, NY: National Tuberculosis and Respiratory Disease Association.

[29] Conde MB, Melo FA, Marques AM, Cardoso NC, Pinheiro VG, et al (2009) III Brazilian Thoracic Association Guidelines on tuberculosis. Jornal brasileiro de pneumologia: publicacao oficial da Sociedade Brasileira de Pneumologia e Tisilogia 35: 1018–1048. 19918635 10.1590/s1806-37132009001000011

[30] Saúde MD (2002) Controle Da Tuberculose: Uma Proposata de Integraçao Ensino-Serviço. 5a Ediçao ed. Rio de Janeiro.

[31] Menzies D (1999) Interpretation of repeated tuberculin tests. Boosting, conversion, and reversion. American journal of respiratory and critical care medicine 159: 15–21. 9872812 10.1164/ajrccm.159.1.9801120

[32] van Zyl-Smit RN, Pai M, Peprah K, Meldau R, Kieck J, et al (2009) Within-subject variability and boosting of T-cell interferon-gamma responses after tuberculin skin testing. American journal of respiratory and critical care medicine 180: 49–58. 19342414 10.1164/rccm.200811-1704OC

[33] Hirji KF MC, Patel NR (1987) Computing Distributions for Exact Logistic Regression JASA. 82: 1110–1117.

[34] Hertzberg G (1957) The infectiousness of human tuberculosis; an epidemiological investigation. Acta tuberculosea Scandinavica Supplementum 38: 1–146. 13424402

[35] Loudon RG, Spohn SK (1969) Cough frequency and infectivity in patients with pulmonary tuberculosis. The American review of respiratory disease 99: 109–111. 5762102 10.1164/arrd.1969.99.1.109

[36] Riley RL, Mills CC, O’Grady F, Sultan LU, Wittstadt F, et al (1962) Infectiousness of air from a tuberculosis ward. Ultraviolet irradiation of infected air: comparative infectiousness of different patients. The American review of respiratory disease 85: 511–525. 14492300 10.1164/arrd.1962.85.4.511

[37] Riley RL, O’Grady F (1961) Airborne infection. New York, NY: Macmillan.

[38] Rich A (1951) The influence of the number of bacilli. In: Thomas C, editor. The Pathogenesis of Tuberculosis. Springfiled. pp. 622–711.

[39] Yoder MA LG, Bishai WR (2004) Cavitary pulmonary tuberculosis: The Holy Grail of disease transmission. Current Science 86: 74–81.

[40] Woodring JH, Vandiviere HM, Fried AM, Dillon ML, Williams TD, et al (1986) Update: the radiographic features of pulmonary tuberculosis. AJR American journal of roentgenology 146: 497–506. 3484866 10.2214/ajr.146.3.497

[41] Sutinen S (1968) Evaluation of activity in tuberculous cavities of the lung. Scand J Respir Dis Suppl 67: 5–78. 4978616

[42] Canetti G (1955) The Tubercle Bacillus in the Pulmonary Lesion of Man.. New York, NY: Springer Publishing Company, Inc.

[43] Rich AR (1951) The Pathogenesis of Tuberculosis, Second ed. Sprinfiled, IL.: Charles C. Thomas.

[44] Perrin FM, Woodward N, Phillips PP, McHugh TD, Nunn AJ, et al (2010) Radiological cavitation, sputum mycobacterial load and treatment response in pulmonary tuberculosis. The international journal of tuberculosis and lung disease: the official journal of the International Union against Tuberculosis and Lung Disease 14: 1596–1602. 21144246

[45] Krishnan N, Malaga W, Constant P, Caws M, Tran TH, et al (2011) Mycobacterium tuberculosis lineage influences innate immune response and virulence and is associated with distinct cell envelope lipid profiles. PloS one 6: e23870. 21931620 10.1371/journal.pone.0023870 PMC3169546

[46] Mathema B, Kurepina N, Yang G, Shashkina E, Manca C, et al (2012) Epidemiologic consequences of microvariation in Mycobacterium tuberculosis. The Journal of infectious diseases 205: 964–974. 22315279 10.1093/infdis/jir876 PMC3415951

[47] Victor TC, Streicher EM, Kewley C, Jordaan AM, van der Spuy GD, et al (2007) Spread of an emerging Mycobacterium tuberculosis drug-resistant strain in the western Cape of South Africa. Int J Tuberc Lung Dis 11: 195–201. 17263291

[48] Albanna AS, Reed MB, Kotar KV, Fallow A, McIntosh FA, et al (2011) Reduced transmissibility of East African Indian strains of Mycobacterium tuberculosis. PloS one 6: e25075. 21949856 10.1371/journal.pone.0025075 PMC3176299

[49] Weisenberg SA, Gibson AL, Huard RC, Kurepina N, Bang H, et al (2012) Distinct clinical and epidemiological features of tuberculosis in New York City caused by the RD(Rio) Mycobacterium tuberculosis sublineage. Infection, genetics and evolution: journal of molecular epidemiology and evolutionary genetics in infectious diseases 12: 664–670. 10.1016/j.meegid.2011.07.018 PMC3290718 21835266

[50] Almeida AS, Lago PM, Boechat N, Huard RC, Lazzarini LC, et al (2009) Tuberculosis is associated with a down-modulatory lung immune response that impairs Th1-type immunity. Journal of immunology 183: 718–731. 10.4049/jimmunol.0801212 19535630

[51] Kato-Maeda M, Gagneux S, Flores LL, Kim EY, Small PM, et al (2011) Strain classification of Mycobacterium tuberculosis: congruence between large sequence polymorphisms and spoligotypes. The international journal of tuberculosis and lung disease: the official journal of the International Union against Tuberculosis and Lung Disease 15: 131–133. PMC3600895 21276309

[52] Whalen CC, Zalwango S, Chiunda A, Malone L, Eisenach K, et al (2011) Secondary attack rate of tuberculosis in urban households in Kampala, Uganda. PloS one 6: e16137. 21339819 10.1371/journal.pone.0016137 PMC3038854

[53] Jones-Lopez EC, Namugga O, Mumbowa F, Ssebidandi M, Mbabazi O, et al (2013) Cough aerosols of Mycobacterium tuberculosis predict new infection: a household contact study. American journal of respiratory and critical care medicine 187: 1007–1015. 23306539 10.1164/rccm.201208-1422OC PMC3707366

