# Supplementary material for: Importance of Cough and M. tuberculosis Strain Type as Risks for Increased Transmission within Households
Source: PLoS One. 2014 Jul 2;9(7):e100984. doi: 10.1371/journal.pone.0100984 (PMC4079704; doi:10.1371/journal.pone.0100984)
Supplement: Table S1 — Additional characteristics of index tuberculosis cases, household contacts, and study dwellings at baseline by Mycobacterium tuberculosis transmission category. 1n (%) or median [IQR]. 2K = Kruskal-Wallis, J = Jonckheere-Trepstra, T = Cochran-Armitage Trend, S = Score test from generalized estimating equation (GEE); W = Wilcoxon rank sum test Missing data: BMI (2). (DOCX) [file pone.0100984.s001.docx]

**Table S1:** Additional characteristics of index tuberculosis cases, household contacts, and study dwellings at baseline by *Mycobacterium tuberculosis* transmission category.

| Characteristic ^1^ | | | | Final Household transmission category | | | | P value ^2^ (Multinomial) | P value ^2^ (LT vs. HT) |
| --- | --- | --- | --- | --- | --- | --- | --- | --- | --- |
|  |  |  |  | **Total** | **Low (LT)** | **Intermediate** | **High (HT)** |  |  |
| *Index Cases* | | | | *N=124* | *N=23* | *N=28* | *N=73* |  |  |
| Body Mass Index (Kg/m²) | | | | 20 [18, 23] | 20 [18, 22] | 20 [18, 23] | 20 [18, 22] | 0.78K | 0.48W |
| Karnofsky performance score | | | | 90 [90, 90] | 90 [85, 90] | 90 [90, 90] | 90 [90, 90] | 0.75K | 0.62W |
| Number of habitable rooms | | | | 5 [4, 7] | 5 [4, 7] | 6 [5, 6] | 5 [4, 6] | 0.24J | 0.50T |
| *Household Contacts* | | | | *N=731* | *N=132* | *N=183* | *N=416* |  |  |
| Number of contacts in past 3 months: median (range) | | | | 5 [4, 7.5] | 5 [4, 7] | 6 [5, 8] | 5 [4, 7] | 0.74J | 0.66W |
| Average exposure time to index case last 3 months (hours/day) | | | |  |  |  |  | 0.30S | 0.41S |
|  | | < 7 | | 247 (34.4) | 38 (28.8) | 50 (29.8) | 159 (38.0) |  |  |
|  | | 7-12 | | 190 (26.5) | 45 (34.1) | 50 (29.8) | 95 (22.7) |  |  |
|  | | 13-18 | | 192 (26.7) | 31 (23.5) | 41 (24.4) | 120 (28.7) |  |  |
|  | | >18 | | 89 (12.4) | 18 (13.6) | 27 (16.1) | 44 (10.5) |  |  |
| Sleeping arrangement | | | |  |  |  |  | 0.35S | 0.51S |
|  | Same House | | |  |  |  |  |  |  |
|  | | | Same room, same bed | 107 (14.9) | 16 (12.1) | 20 (11.9) | 71 (17.0) |  |  |
|  | | | Same room, different bed | 61 (8.5) | 14 (10.6) | 17 (10.1) | 30 (7.2) |  |  |
|  | | | Different room | 313 (43.6) | 61 (46.2) | 79 (47.0) | 173 (41.4) |  |  |
|  | Different house | | | 237 (33.0) | 41 (31.1) | 52 (31.0) | 144 (34.5) |  |  |

^1^ n (%) or median [IQR]

^2^ K=Kruskal-Wallis, J=Jonckheere-Trepstra, T=Cochran-Armitage Trend, S=Score test from generalized estimating equation (GEE); W=Wilcoxon rank sum test

Missing data: BMI (2)
